# Attentional Bias for Emotional Information in Older Adults: The Role of Emotion and Future Time Perspective

**DOI:** 10.1371/journal.pone.0065429

**Published:** 2013-06-04

**Authors:** Ineke Demeyer, Rudi De Raedt

**Affiliations:** Department of Experimental Clinical and Health Psychology, Ghent University, Ghent, Belgium; George Mason University/Krasnow Institute for Advanced Study, United States of America

## Abstract

**Objectives:**

Research suggests that older adults display a positivity bias at the level of information processing. However, because studies investigating attentional bias for emotional information in older adults have produced mixed findings, research identifying inter-individual differences that may explain these inconsistent results is necessary. Therefore, we investigated whether mood, symptoms of depression, symptoms of anxiety and future time perspective are related to attentional bias in older adults.

**Method:**

Thirty-seven healthy older adults and 25 healthy middle-aged adults completed questionnaires to assess mood, symptoms of depression, symptoms of anxiety and future time perspective. Attentional bias towards happy, sad and neutral information was measured using a modified exogenous cueing paradigm with long cue presentations, to measure maintained attention versus avoidance of emotional stimuli.

**Results:**

Older adults showed attentional avoidance for all emotional faces, whereas no attentional biases were found in the middle-aged group. Moreover, in the older adult group, avoidance for negative information was related to anxiety. Future time perspective was unrelated to attentional bias.

**Discussion:**

These findings suggest that anxiety may lead to inter-individual differences in attentional bias in older adults, and that avoidance from negative information may be an emotion regulation strategy.

## Introduction

Although older adults are increasingly confronted with negative events, such as loss of significant others, there seems to be no increase in their experience of negative affect. On the contrary, even though mixed results were reported, there is evidence that aging tends to be characterized by a decrease in negative affect and by a stabilization or even an increase in positive affect [1], [2]. It has been suggested that this paradox could be explained by an improvement in emotion regulation [3]. The socioemotional selectivity theory [4] provides a framework for understanding the optimization of emotion regulation in older adults. According to this theory, the way people perceive their remaining time in life influences motivation and goal preferences. Because future time perspective becomes more limited as people get older, older adults would prioritize present-oriented goals of emotional well-being. This shift towards emotional goals would lead to changes in information-processing tendencies. More specifically, congruent to the goals of emotional well-being, an increased preference towards positive information and/or away from negative information emerges. This is known as the ‘positivity effect’ [5] and it has been proposed that this would enhance emotional well-being.

Studies examining the socioemotional selectivity theory have investigated age-related differences in emotional information processing in search for a positivity effect in older adults. Even though several studies demonstrated superior memory for positive material in older adults compared to younger adults (for a review, see [6]), findings about a positivity effect in attention were less conclusive. Some studies found that older adults showed an attentional bias towards positive information that was not present in younger adults (e.g. [7]). Other studies demonstrated that younger and older adults only differed in attentional bias towards negative stimuli and concluded that the positivity effect in older adults is driven by a focus away from negative information (e.g. [8]). Although several studies reported evidence for some form of positivity bias in older adults, not all studies could confirm this. A recent study [9] found no difference between young and older adults using a rapid serial visual presentation task and concluded that emotion influences attention of both age groups in the same way. Moreover, Murphy and Isaacowitz [10] conducted a large meta-analysis which involved 1085 older adults and 3150 younger adults who participated in studies investigating either attention or memory for emotional stimuli. Few overall age differences were found, leading the authors to conclude that emotional information processing would remain stable across adulthood.

So far, studies investigating information processing in older adults mainly compared older to younger adults (e.g. [8], [10]). Given the inconsistent findings in older adults, research taking a closer look at within-group differences that might explain these conflicting results is necessary. To increase our understanding of the positivity effect in older adults, we need to identify underlying factors that may influence attentional bias in older adults. Recently, there have been studies uncovering factors that may influence the positivity effect, such as dispositional cognitive reappraisal [11] and interdependent self-construal [12]. Based on the literature, we argue that also emotions and future time perspective may play a role.

There is a general consensus regarding the influence of emotions on cognitive processing. Even though empirical evidence in younger groups points towards a relationship between affective disorders and preferential processing of negative material (e.g. [13], [14]), mood and affective symptoms are usually ignored in older adult studies. Research in younger-aged adults has shown that depression is characterized by a bias towards negative information at later stages of information processing (for a review, see [15]) and that anxiety is associated with a bias towards threatening information (for a review, see [16]). However, little work has been done to investigate whether the positivity effect in information processing in older adults relates to inter-individual differences in mood and affective symptoms. Although it has been suggested that emotional well-being would increase with age, not all studies have confirmed this. Moreover, recent studies pointed out that the prevalence of depressive symptoms remains high in late life and that symptoms of anxiety are even more common (for a review, see [17]). Studies in older adults with anxiety disorder demonstrate a bias towards negative information [18]. Furthermore, it has been demonstrated that also healthy older adults with elevated fear or worry show attentional bias towards fear-relevant or threatening stimuli [19], [20], [21]. Recently, Orgeta [22] showed that older adults who experience more anxiety also report more difficulties in regulating their emotional experiences. Therefore, it is possible that inter-individual differences in mood and non- clinical symptoms of anxiety and depression in older adults may influence information processing and hamper the positivity effect, which might explain the inconsistent results in the older adult literature.

A second factor that might influence information processing is introduced by the socioemotional selectivity theory [4]. As mentioned above, this theory assumes that future time perspective is a crucial factor leading to a shift in goals and changes in information processing. So far, studies investigating this issue mainly investigated differences in information processing between age groups [8], [10], assuming a different future time perspective between age groups without measuring this variable. Importantly, the theory states that, even though age is related to future time perspective, individuals are able to adopt a future time perspective that is not in line with their chronological age. Moreover, studies have shown that the same motivational changes can occur in other contexts than ageing (e.g. [23]) and that inter-individual differences in future time perspective are also present within older adult groups [24]. Therefore, future time perspective may be a more important factor than age for the positivity effect to emerge, and inter-individual differences in future time perspective in older adults may account for the inconsistent findings on attentional bias in older adults.

### The Current Study

The first aim of our study was to investigate whether we could replicate findings of prior studies that showed differences in attentional bias between younger and older adults. It has been argued that the positivity effect can mainly be observed in the oldest cohorts [6]. Therefore, only participants older than 75 were included in our older adult group. Previously mentioned research has mainly compared older adults (>60 years) with young undergraduate samples (<30 years). Because these studies cannot conclude whether the changes in information processing occur already at middle-age or only in late-life such as theorized, we selected a middle-aged adult group between the age of 27 and 55 as a more appropriate comparison group.

To examine attentional bias, we used an emotional variant of the exogenous cueing task [25]. In line with previous studies using this task (e.g. [14]), pictures of faces were selected as emotional cues. More specifically, we used pictures of neutral faces to establish a baseline and pictures of happy and sad faces to investigate attentional bias for positive and negative information. We selected sad faces, because previous studies found no age-related differences using threatening stimuli. To explain these findings, Mather and Knight [26] suggested that processing of threatening information remains crucial for survival and is unlikely to be influenced by emotional goals. Lately, it has been assumed that the positivity effect in older adults might only occur in later stages of top-down processing and not in early automatic processing [6], [9]. Fast automatic processing would be inaccessible to attentional control, which would prevent influences by emotional goals. More precisely, Isaacowitz, Allard, Murphy and Schlangel [27] demonstrated that the positivity effect in older adults only appeared 500 ms and later after stimulus onset. In light of these findings, we opted for long cue presentations (1000 ms) in the exogenous cueing task. Based on the socioemotional selectivity theory, we predicted that older adults would show more maintained attention towards positive information and/or more avoidance of negative information as compared to middle-aged adults, even when controlling for a possible decline in processing speed in older adults.

The second aim of this study was to investigate the relationship between attentional bias, mood and affective symptoms in older adults because the influence of emotions has been understudied in this age group. In the present study, we included measurements of mood (PANAS), symptoms of anxiety (STAI) and depression (BDI). Based on the younger adult literature, we expected that in both middle-aged and older adults more negative mood/affective symptoms would be related to less maintained attention for positive information and more maintained attention for negative information.

The third aim of this study was to investigate the direct link between future time perspective and attentional bias. Therefore, we included a measure of future time perspective. Given that future time perspective can have an influence independent of age [23], we hypothesized that both middle-aged and older adults with a more limited future time perspective would show more maintained attention for positive information and more avoidance of negative information.

## Methods

### Participants

The current study was approved by the ethical committee of the faculty of psychology and educational sciences of Ghent University. Forty-five older adults were recruited through several organizations for older adults. All participants were active, independently living older adults with no current psychiatric disorder as assessed with the Mini International Neuropsychiatric Interview (MINI; [28]). Participants who made too many errors or outliers (see data preparation) on the task were excluded from the study. The final sample consisted of 37 participants (23 women, 14 men) between the ages of 75 and 88 (*M = *78.57; *SD = *3.59). In this group, 57% was married, 35% was widow/widower and 8% was single.

The middle-aged group consisted of 25 adults (15 Women, 10 men) ranging in age from 27 to 55 years (*M = *45.20; *SD = *8.01). Based on the MINI, participants had no psychiatric disorders. Moreover, 88% was married and 12% was single.

### Materials

#### Affective well-being measures

The Beck Depression Inventory (BDI-II; [29]) was used to measure the presence and the degree of depressive symptoms. The trait version of the State-Trait Anxiety Inventory (STAI; [30]) was administered to index anxiety and proneness to respond anxiously to stressful situations. To assess mood, the trait version of the positive and negative affect schedule (PANAS; [31]) was used. We used the Dutch versions of these questionnaires, which have shown good psychometric properties (respectively, [32], [33], [34]). Moreover, all these questionnaires demonstrated acceptable to good reliability in both the older (all Cronbach’s Alpha >.74) and middle-aged adult group (all Cronbach’s Alpha >.79).

#### Future Time Perspective Scale (FTPS)

The extent to which participants see their future as expansive was assessed with a Dutch translation (by the authors) of the FTPS by Carstensen and Lang (1996, unpublished manuscript). Participants rate their agreement with 10 statements on a 7-point scale. Higher scores indicate a more expansive perception of the future. The Dutch translation of the FTPS has shown acceptable psychometric qualities (unpublished data). In our samples, Cronbach’s Alpha was.67 for older adults and.78 for middle-aged adults.

#### Exogenous Cueing Task (ECT)

The exogenous cueing task was programmed in INQUISIT Millisecond software. As cues, 60 coloured pictures of emotional faces were selected from the Karolinska Directed Emotional Faces database (KDEF). This selection was based on prior validation [35]. Twenty happy, 20 neutral and 20 sad facial expressions were included based on correct categorisation (>90% for happy, >85% for sad, >80% for neutral) and average rating on a 9-point Likert scale of how well the picture reflects the emotion (M = 5.28 for neutral, M = 6.78 for happy, M = 6.02 for sad). All pictures were cut to exclude interference of background stimuli (hair, clothing), and they were adjusted to the same size (326×326 pixels). The ability of older adults to correctly recognize these emotional stimuli was crucial for this study. Therefore, the stimuli were also rated by a subsample (N = 18) of the older adult group. After the complete experiment, older adults were asked to rate a subset (20 pictures) of the stimuli. The percentage of correct identification was 96% for happy faces, 93% for neutral faces and 85% for sad faces. Moreover, they also rated how well the picture reflected the emotion on a 9 point scale. Average ratings were 7.01 for happy, 6.06 for neutral, and 6.04 for sad faces.

All participants were seated at 60 cm viewing distance of the computer screen (a 19-inch colour monitor). Stimuli were presented against a black background. Each trial started with the presentation of two white frames (75 mm by 75 mm, visual angle: 7.15°) located on both sides of a fixation cross. These remained on screen throughout the entire trial. The middle of each of these frames was at 40 mm distance (3.81° visual angle) from the fixation cross. Exactly 500 ms later, the cue appeared for 1000 ms, replacing one of the frames. Immediately after the cue disappeared, a target (a black square, 10 mm by 10 mm, visual angle 1°) was presented in the middle of one of the two frames and remained on screen until response. Participants were instructed to indicate as quickly and accurately as possible the location of this target by pressing on the left or right button of a response box. It was emphasized that attention should be directed towards the fixation cross during the entire experiment. Participants got acquainted with the exogenous cueing task during 16 practice trials. Subsequently, they performed the test block, which consisted of 240 trials.

The location of the picture cued the location of the target correctly on 50% of the trials (valid trials) and incorrectly on the other 50% (invalid trials). Participants were informed that the location of the cue was not predictive for the target location. All the pictures were presented randomly with an equal number of presentations and trial type (valid versus invalid). Using long cue presentations, people can be faster at responding to invalid trials in comparison to valid trials. This effect is known as the inhibition of return (IOR) effect [36] and results from inhibition of the previously attended location in favor of the unattended location.

To control for response strategies (for example focussing on only one frame during the experiment), 24 trials were inserted in which the fixation cross was briefly (150 ms) replaced by an arrow. Participants had to indicate if this arrow pointed left or right. Three participants were removed from analysis due to their mistakes (more than 50%) on these arrow trials.

### Data Preparation

Trials with errors (*M = *2%) were omitted from analyses. Based on a boxplot, responses shorter than 200 ms and longer than 1250 ms were considered to be outliers (*M = *6.9%). They respectively reflect anticipatory and delayed responding and were also discarded from further analyses. No significant differences were found in the emotional valence associated with the errors or outliers (all *p*>.52). Five older adults were excluded because of a loss of more than 25% of their data. Statistical analyses were performed on the remaining data.

### Procedure

Participants were tested individually in a quiet environment at their home. The older adults were tested in the morning because this time of day results into their most optimal performance [37]. At the beginning of the experiment, written informed consent was obtained and participants started the ECT task. Halfway the task, participants were offered the possibility to take a break. Participants completed the questionnaires at the end of the attention task to avoid emotional priming. At the end, all participants were debriefed.

## Results

### Group Characteristics

Table 1 gives an overview of mean scores, standard deviations and correlations for all questionnaires. As expected, a significant difference was found between age groups for the FTPS, *t*(60) = 7.76, *p*<.01, indicating that older adults showed a more limited future time perspective as compared to middle-aged adults. With respect to negative affect (PANAS) and anxiety (STAI), older adults did not differ significantly (all *t*s<1.4) from the middle-aged adults. However, a significant difference was found for positive affect (PANAS), *t*(60) = 2.92, *p*<.01, and for BDI, *t*(60) = 3.05, *p*<.01, with older adults showing less positive affect and more depressive symptoms than middle-aged adults. Future time perspective did not correlate with other questionnaires in the older adult group (all *r*<.23, *p*>.17). In the middle-aged group, the FTPS was negatively correlated to the BDI, *r*(25) = −.50, *p*<.05, which indicates that a more limited future time perspective was related to more depressive symptoms.

**Table 1:** Mean scores, standard deviations and zero-order correlations for middle-aged and older adults on all the questionnaires.

	older adults						middle-aged adults					
	M	SD	(1)	(2)	(3)	(4)	M	SD	(1)	(2)	(3)	(4)
(1) FTPS	27.62	7.12	–				42.48	7.80	–			
(2) PA	32.14	6.67	−.18	–			36.44	3.80	.28	–		
(3) NA	16.32	4.04	.02	−.27	–		17.12	4.88	−.19	−.20	–	
(4) BDI	6.19	4.18	.22	−.35*	.43**	–	3.04	3.69	−.50*	−.27	.42*	–
(5) STAI	33.59	6.88	.23	−.46**	.55**	.70**	36.12	6.70	−.36	−.18	.75**	.58**

* Correlation is significant at the 0.05 level (2-tailed).

** Correlation is significant at the 0.01 level (2-tailed).

### Group Differences on the Exogenous Cueing Task

The reaction times on the attention task were subjected to a 3×2×2 mixed ANOVA (multivariate approach) with Cue Valence (happy, neutral, sad) and Trial Validity (valid, invalid) as within subjects variables and Group (older and middle-aged adults) as between subjects variable. Mean reaction times and standard deviations are presented in table 2. There was a significant main effect of Trial Validity, *F*(1,60) = 13.05, *p*<.01, η^2^_p = _.18. A significant effect was also found for Group × Trial Validity, *F*(1,60) = 19.19, *p*<.01, η^2^_p = _.24. More importantly, there was a significant three-way Group × Cue Valence × Trial Validity interaction, *F*(2,59) = 3.99, *p*<.05, η^2^_p = _.12. Given that aging is known to be accompanied with a decline in cognitive processing speed leading to slower response latencies in older adults compared to younger adults, we repeated these analyses with linearly transformed reaction times as recommended by Faust, Balota, Spieler, & Ferraro [38]. For each condition mean RTs were converted to z-scores by subtracting each participant’s mean RT per condition from his overall mean RT, which was divided by the standard deviation of the mean of that condition. These converted reaction times were also subjected to a 3×2×2 ANOVA. If anything, the three-way interaction effect of Group × Cue Valence × Trial Validity became even more significant (*F*(2,59) = 4.830, *p*<.05). Thus, these results demonstrated that the age-related cognitive slowing is not causing the difference in attentional bias between older and middle-aged adults.

**Table 2:** Mean reaction times (in ms) and standard deviations (SD) as a function of Trial Validity and Cue Valence in middle-aged and older adults.

		Older adults		Middle-aged adults	
Cue valence	Trial Validity	M	SD	M	SD
happy	valid	598	123	379	61
	invalid	563	125	387	51
neutral	valid	581	124	384	60
	invalid	564	127	383	45
sad	valid	593	122	383	59
	invalid	557	128	385	51

To further investigate the significant three-way interaction effect, a 3×2 ANOVA was performed for both groups separately. No significant effects were found in the middle-aged group (all *F*<2.5, *p*>.05). In the older adult group, a significant main effect for Trial Validity, *F*(1,36) = 30.96, *p*<.01, η^2^_p = _.46, was found with overall faster responding on invalid trials in comparison with valid trials (the IOR effect). The ANOVA for older adults also revealed the crucial significant interaction effect between Cue Valence and Trial Validity, *F*(2,35) = 3.72, *p*<.05, η^2^_p = _.18.

To further clarify this effect, Cue Validity Indexes (CVI) were calculated (RT invalid cue - RT valid cue) for each emotion. As mentioned above, using long cue presentations, people can be faster at responding to invalid trials in comparison to valid trials. The magnitude of this inhibition of return effect [36] points to the degree of avoidance of the cue (versus maintained attention). In this way, the more negative the CVI scores, the more attentional avoidance. It may be expected that emotionally relevant stimuli will elicit less inhibition of return compared to neutral information. In this case, a reduced inhibition of return for emotional stimuli compared to neutral stimuli indicates maintained attention [39]. The opposite finding (e.g. more IOR for emotional compared to neutral stimuli) indicates attentional avoidance.

**Figure 1:**
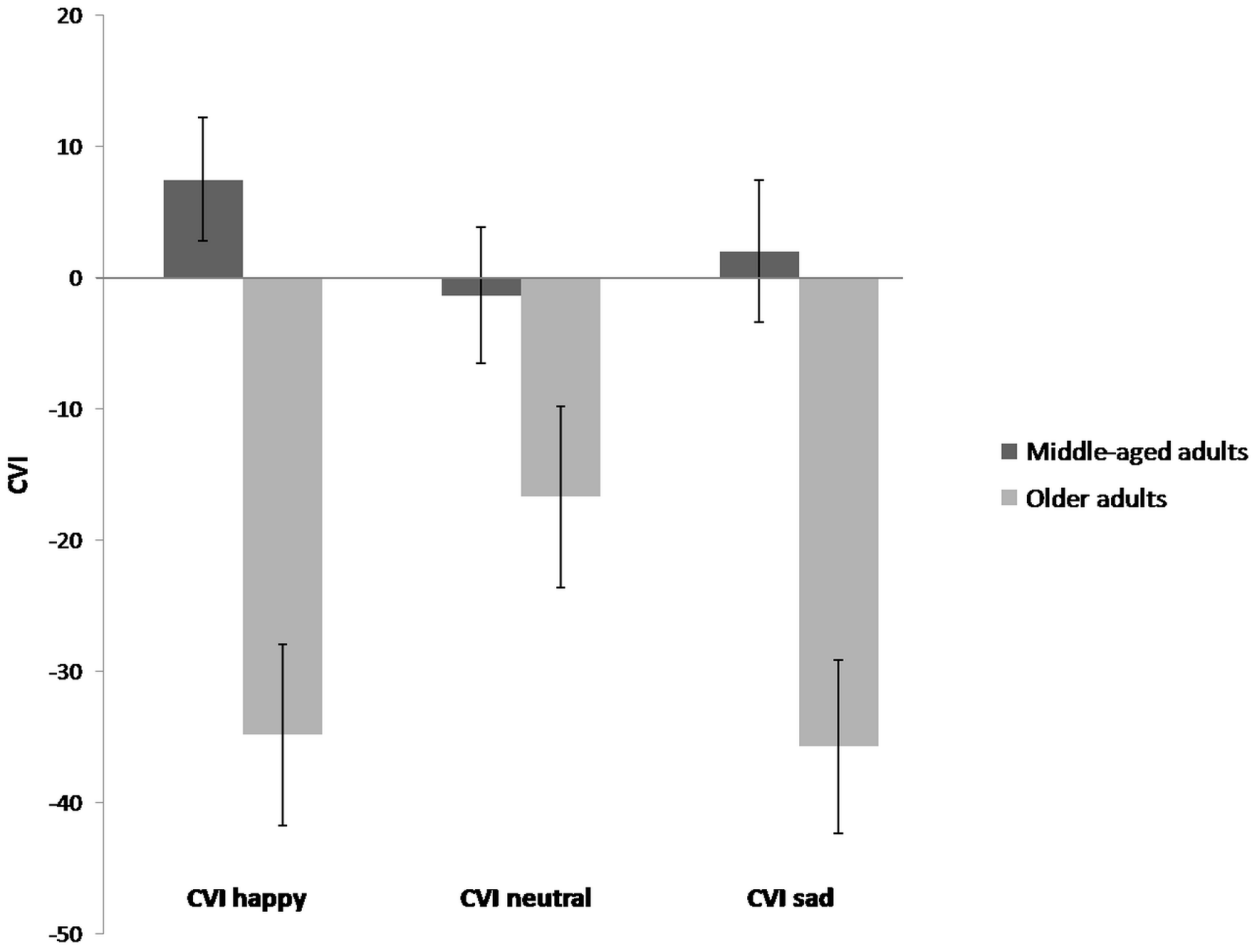
The Cue Validity Indexes for happy, neutral and sad information in middle-aged and older adults.

Using paired samples t-tests, we observed a significant difference between the CVI for both happy, *t*(36) = 2.39, *p*<.05, and sad faces, *t*(36) = 2.46, *p*<.05, as compared to the CVI for neutral faces in the older adult group. However, there was no difference between the CVI for sad and happy faces (*t*<1). This effect shows more inhibition of return (faster on invalid as compared to valid trials) for both emotional expressions as compared to neutral expressions (the baseline) as shown in figure 1. Thus, there seems to be more avoidance for all emotional stimuli.

Moreover, the CVI scores were also used to further investigate the differences between middle-aged and older adults. As expected, the groups did not differ on the CVI for neutral faces (*t* = 1.63, *ns*) using independent samples t-tests. However, the difference between groups was significant for both the CVI for happy faces, *t*(60) = 4.57, *p*<.01, and the CVI for sad faces, *t*(60) = 4.09, *p*<.01, indicating more inhibition of return for both emotional stimuli in the older adult compared to the middle-aged group.

### Attentional Bias, Mood and Symptoms of Anxiety and Depression

The second aim of the study was to investigate the link between attention and mood/affective symptoms. Therefore, we first investigated differences between the middle-aged and older adult group by using the 4 measures of mood and affective symptoms (positive affect, negative affect, BDI and STAI) as continuous independent variables (covariates) in 4 separate 3×2×2 mixed ANOVA (multivariate approach) with Cue Valence and Trial Validity as within subjects variables and Group as between subjects variable. Significant 4-way interaction effects were only found using the STAI as covariate, *F*(2,57) = 5.04, *p = *.01, η^2^_p = _.15 (all other *F*<2.58, *p*>.05). To further investigate the effects, the reaction times were also subjected to 4 separate 3 (Cue valence) × 2 (Cue Validity) ANOVAs with mood and affective symptoms as covariates for both age groups separately. In the middle-aged group, there were no significant interaction effects with all 4 measures of mood and affective symptoms (all other *F*<.88, *p*>.05). In the older adult group, the 3 ANOVAs using positive affect, negative affect and BDI as covariate yielded no significant interaction effects (all *F*<2.50, *p*>.10) that could point to a relation between mood/affective symptoms and attentional bias. However, when anxiety symptoms, as measured by the STAI, were used as a covariate in the ANOVA a significant 3-way interaction effect was found, *F*(2,34) = 10.07, *p*<.001, η^2^_p = _.37. Given that 4 separate ANOVAs were used, it is important to emphasize that this effect survives Bonferroni corrections. The same analyses were repeated in the middle-aged group, but no significant effects were found (all *F*<2.47, *p*>.10).

To follow-up on the significant interaction effect in older adults, Pearson correlation coefficients between the STAI and attentional bias indices were calculated. Using CVI for neutral faces as a baseline, 2 new variables were constructed by subtracting CVI for neutral from both CVI for happy faces and CVI for sad faces. In the older aged group, CVI (sad - neutral) was significantly correlated with STAI scores, *r*(35) = −.60, *p*<.001. These results indicate that older adults who reported more symptoms of anxiety also showed more avoidance for sad information. No significant correlations were found with CVI (happy – neutral).

### Attentional Bias and Future Time Perspective

The third aim of the study was to investigate the link between future time perspective and attentional bias in both age groups. The FTPS was used as a continuous independent variables (covariate) in a 3 (Cue Valence) ×2 (Cue Validity) ×2 (Group) ANOVA. This analyses yielded no significant interaction effects (all *F*<2.78, *p*>.05). When further inspecting the data by subjecting it to 2 separate 3 (Cue valence) ×2 (Cue Validity) ANOVAs for both age groups with future time perspective as covariate, we found no significant effects within both groups (all *F*<2.05, *p*>.15) between future time perspective and attentional bias.

## Discussion

This study examined attentional bias at later stages of information processing in older and middle-aged adults. Moreover, given inconsistent findings in previous research, we investigated whether inter-individual differences in attentional bias were related to mood, affective symptoms and future time perspective. Our findings showed a difference in attentional bias in older adults aged over 75 compared to a sample of adults between the age of 27 and 55. This adds to the evidence for a late-life-change in information processing because previous research mostly compared older adults to young undergraduate samples. Consistent with previous research (e.g. [8]), we found no attentional bias in the middle-aged group. This might be linked to their rather low scores on the negative affect measures. When investigating the age-related differences in more detail, we found that the middle-aged group differed from the older adult group for both the Cue Validity Index for happy and sad faces, but not for neutral faces, indicating that, in line with our expectations, there was no difference between the age groups in attentional bias for non-emotional information.

In contrast to middle-aged adults, older adults displayed attentional bias for emotional information in comparison to neutral information. However, contrary to expectations based on the socioemotional selectivity theory, they showed more avoidance for all emotional information and not only for negative faces. One explanation for this difference of our results compared to previous research might be related to our attention paradigm. Importantly, this paradigm does not allow to make inferences about initial attentional capture. However, compared to previously used paradigms, the exogenous cueing task with longer presentation times can specifically measure maintained attention or attentional avoidance. It has been suggested that attentional avoidance at later stages of information processing is based on emotion regulation goals [16] and that older adults would use more passive emotion regulation strategies like avoidance, to protect themselves from arousal and to maintain energy [40]. Accordingly, it could be argued that by avoiding all emotional information, even positive information, older adults attempt to regulate and to maintain a constant level in their emotional state. Moreover, compared to previous studies, our older adult sample was older (75 to 88 years), which might also have contributed to the differences found in affective well-being and attentional bias. However, based on the socioemotional selectivity theory, the positivity effect should increase with ageing because the older people get, the more they are confronted with an even more limited future time perspective.

The second purpose of this study was to investigate whether inter-individual differences in mood and affective symptoms are related to attentional bias. Our results demonstrated that the age groups differed in how attentional bias was related to both negative affect and anxiety. In contrast to previous studies that usually report attentional bias towards mood-congruent information [13], we found no significant indications of a link between mood and attentional bias in middle-aged adults. In light of these findings it is important to remark that the middle-aged group reported very little negative mood/affective symptoms, which may have prevented us from finding any significant effects. More importantly, we found that older adults who experienced more anxiety symptoms showed a larger IOR effect for sad compared to neutral stimuli, which is indicative of more attentional avoidance of sad stimuli. No results were found with the other affect measures. In contrast to younger adults who usually display attentional bias towards mood-congruent information [13], older adults with elevated anxiety levels showed attentional bias away from negative information. These results contradict previous studies that point towards a vigilance for negative information in anxious adults [19]; [20]; [21]. However, these studies mainly used fear-relevant stimuli and it has already been argued that processing of threatening information may remain crucial over the whole life-span [26] and that attentional processing of older adults may vary over the type of stimuli used [41]. Using similar stimuli as the current study, Lee and Knight [41] also found avoidance of sad faces in a later stage of attentional processing in older adults at moderate levels of anxiety. In line with Isaacowitz, Toner, Goren, and Wilson [42] who demonstrated that gaze preference towards positive information is most apparent in older adults confronted with negative affect, we argue that anxiety in older adults might signal that emotion regulation is needed, and motivates to activate the avoidance strategy.

In general, avoiding negative information might be seen as functional mechanism to maintain a neutral/positive emotional state. However, it has also been argued that frequent use of avoidance can be seen as a maladaptive emotion regulation strategy, preventing emotional processing of the information and increasing risk for several psychological disorders, such as anxiety and depression [43], [44]. In a former study using exactly the same paradigm, but with death related cues included, we observed less attentional avoidance of threat in older adults as compared to younger adults, suggesting that less avoidance might mean that death becomes less of a concern in older adults [45]. In line with these findings, our results might indicate that avoidance of negative information is a maladaptive form of emotion regulation in older adults because it coincides with higher levels of anxiety. However, given the cross-sectional data obtained here, we cannot state whether anxiety interferes with adaptive emotion regulation resulting in avoidance for negative information or whether this avoidance leads to anxiety. Future research needs to investigate whether attentional avoidance of negative information points towards a maladaptive emotion regulation strategy in older anxious adults. This may lead to promising improvements in clinical practice, such as identifying older adults at risk for emotional disorders and targeting emotion regulation strategies in the treatment of distress. More importantly, the results of current study emphasize the importance of taking anxiety into account when examining information processing in older adults.

The third purpose of this study was to investigate whether inter-individual differences in future time perspective are related to attentional bias. In contrast to what might be expected based on the socioemotional selectivity theory, no significant relationship between future time perspective and attention was found. In addition to recent findings [2] showing that future time perspective did not influence the relationship between age and affect, this indicates that future time perspective might not be the crucial factor leading to beneficial information processing and more affective well-being. However, more research is necessary because the rather low psychometric properties of the future time perspective scale in the older adult group may have prevented finding significant relationships. Future research might benefit from manipulating future time perspective to further clarify the relationship between future time perspective and attentional processing. Identifying factors that might influence emotionally-beneficial information processing, such as future time perspective, may lead to clinical applications targeting these factors to improve mental health.

Some limitations should be acknowledged. Although our group of older adults consisted of independently living adults, it could have been useful to include a measure of cognitive abilities. Mather and Knight [37] pointed out that older adults with better cognitive functioning showed more positivity bias. It is possible that the older adults, who were omitted from the analyses because of data loss, would also score lower on cognitive abilities. Moreover, the size of our sample may limit the generalizability of the results. Furthermore, our group consisted of healthy older adults who only experienced subclinical levels of anxiety and depression. For future research, it might be interesting to test clinically anxious or depressed older adults. Moreover, based on our hypothesis, we focused on trait measures of affect, but we cannot exclude an impact of mood state during the experiment. Finally, given the correlational data obtained here, we cannot make any statements about the direction of the relationship between anxiety and attention or exclude possible cohort effects.

Notwithstanding these limitations, to our knowledge, this is the first study to investigate whether inter-individual differences in attentional bias within a group of older adults can be explained by mood, affective symptoms and future time perspective. Moreover, our study adds to the existing literature by using a different attention paradigm focusing on maintained attention and avoidance, and by using a middle-aged versus older adult (>75 years) sample. First, we showed that subclinical symptoms of anxiety are related to attentional processing in older adults. Secondly, we included a measurement of future time perspective to investigate its relationship with attentional bias. Although this is one of the basic factors within the socioemotional selectivity theory, few studies have measured future time perspective. Although our results were not in line with the theory and require replication, they hold potential to stimulate further research into the role of future time perspective.

To summarize, we found age-related differences in attentional bias. Compared to the middle-aged group, older adults showed avoidance from both negative as positive information. When taking a closer look into the role of mood and affective symptoms, we found that older adults who experienced more anxiety symptoms showed more avoidance of negative stimuli. Even though future research needs to confirm that attentional avoidance of negative information is an emotion regulation strategy, our results showed that anxiety may lead to inter-individual differences in attentional bias in older adults.
